# Photocatalytic Inactivation of Co-Culture of *E. coli* and *S. epidermidis* Using APTES-Modified TiO_2_

**DOI:** 10.3390/molecules28041655

**Published:** 2023-02-09

**Authors:** Paulina Rokicka-Konieczna, Agnieszka Wanag, Agnieszka Sienkiewicz, Dylan Shun Izuma, Ewa Ekiert, Ewelina Kusiak-Nejman, Chiaki Terashima, Atsuo Yasumori, Akira Fujishima, Antoni W. Morawski

**Affiliations:** 1 Department of Inorganic Chemical Technology and Environment Engineering, Faculty of Chemical Technology and Engineering, West Pomeranian University of Technology in Szczecin, Pułaskiego 10, 70-322 Szczecin, Poland; 2 Department of Pure and Applied Chemistry, Faculty of Science and Technology, Tokyo University of Science, 2641 Yamazaki, Noda, Chiba 278-8510, Japan; 3 Research Center for Space System Innovation, Research Institute for Science and Technology, Tokyo University of Science, 2641 Yamazaki, Noda, Chiba 278-8510, Japan; 4 Department of Materials Science and Technology, Faculty of Advanced Engineering, Tokyo University of Science, 6-3-1 Niijuku, Katsushika-ku, Tokyo 125-8585, Japan

**Keywords:** photocatalysis, titanium dioxide, APTES, antibacterial activity, mixed bacteria cultures

## Abstract

The presented work shows the antibacterial activity of TiO_2_ photocatalysts modified by 3-aminopropyltriethoxysilane (APTES). The APTES-functionalized TiO_2_ samples were obtained by the solvothermal process followed by calcination. The antibacterial activity of APTES/TiO_2_ samples was evaluated with two species of bacteria, *Escherichia coli* and *Staphylococcus epidermidis*, under artificial solar light (ASL) irradiation. The used bacteria are model organisms characterized by negative zeta potential (approx. −44.2 mV for *E. coli* and −42.3 mV for *S. epidermidis*). For the first time, the antibacterial properties of APTES-functionalized TiO_2_ were evaluated against mono- and co-cultured bacteria. The high antibacterial properties characterized the obtained APTES-modified nanomaterials. The best antibacterial properties were presented in the TiO_2_-4 h-120 °C-300 mM-Ar-300 °C sample (modified with 300 mM of APTES and calcined at 300 °C). The improvement of the antibacterial properties was attributed to a positive value of zeta potential, high surface area, and porous volume.

## 1. Introduction

With the continuous development of science and technology and people’s awareness, more and more attention is being paid to aspects of environmental protection. It relates, in particular, to the pollutants present in water systems. Drinking water is one of the most strictly monitored resources as it is controlled by many quality standards [1,2,3]. Nonetheless, water purity is still a scientific and technical challenge due to widespread pathogenic microorganisms that are difficult to remove using conventional disinfection methods (such as chlorination). Moreover, the number of microorganisms resistant to the commonly used antibiotic and antimicrobial agents is also increasing [4,5]. Additionally, aquatic environments are considered ideal for acquiring and disseminating antibiotic resistance, simultaneously representing an additional human health risk [6,7,8,9].

Numerous disinfection systems and technologies characterized by varied effectiveness are currently applied for water treatment. Unfortunately, their applications can be limited to some deficiencies. The most widely used method for disinfecting water is chlorination. However, chlorine may also oxidize natural organic matter (NOM) in water, which leads to the production of unwanted disinfection byproducts (DBPs) [10]. Ozone-based disinfection systems are expensive (they require system equipment with unique corrosion-resistant materials, ozone generation, and use lots of energy) [11]. The use of photo-assisted technologies based, e.g., on UV disinfection, given the risk of bacteria regrowth in the water supply network, usually requires secondary residual disinfectants (such as chlorine as mentioned above) [12]. This requires the search for alternative methods of water disinfection.

Advanced Oxidation Processes (AOPs), including photocatalytic oxidation with TiO_2_, are promising methods that have received much research attention in water treatment [13,14,15]. These processes include various technologies destroying a broad spectrum of organic and inorganic contaminants and undesirable water microorganisms. One of the main advantages of photocatalysis is the efficient degradation of pollutants without any chemical additives. Additionally, it can be used in various applications such as water treatment, air purification, or self-cleaning glass coating. It also is a relatively low-cost method [14,16]. Unfortunately, TiO_2_ exhibits some limitations. For example, due to a large band gap (3.2 eV), TiO_2_ can be activated only by UV light [14]. On the other hand, the rapid recombination of the photogenerated hole–electron pairs reduced TiO_2_ photocatalytic efficiency [17]. Therefore, many attempts have been performed over the past few decades to improve TiO_2_ activity and sensitivity under visible light [18,19].

A literature review reveals that TiO_2_ co-modifications have been presented as a promising and inexpensive choice for improving photocatalytic activity [20,21]. Co-modification improves photocatalytic activity and specific characteristics compared to single-element modification [20]. Many authors reported that incorporating nitrogen or carbon into TiO_2_ materials led to obtaining photocatalysts with enhanced efficiency and activity under UV and visible light [22,23,24]. In turn, adding silica can increase the crystal stability and surface area and enhance the overall TiO_2_ photocatalytic activity [25]. According to Klarysri et al. [26], 3-aminopropyltriethoxysilane (APTES) is one of the most commonly used organic silica sources that can be used for co-modifying the TiO_2_ surface.

Most research on photocatalytic inactivation of microorganisms is carried out under laboratory conditions using pure cultures, single strains, or bacterial species (monocultures). Exceptions include Van Grieken et al. [27], who investigated the photocatalytic inactivation of the mixture of *Escherichia coli* and *Enterococcus faecalis* in a suspension simulating sewage treatment plant leachate. Rincón and Pulgarin [28] examined the photocatalytic disinfection of water polluted by a mixture of *Escherichia coli* and *Bacillus* sp. and wastewater containing a broad bacterial community. Therefore, the impact of TiO_2_ on bacteria co-culture in water is not as well investigated. Since microorganisms such as bacteria form dual and multispecies co-cultures in almost all natural environments, it has been decided to examine the photocatalytic disinfection of water containing a mixed culture of two bacteria. Additionally, our previous works [29,30] showed that the modification of titanium dioxide using APTES resulted in the preparation of the modified photocatalysts, which exhibited good antibacterial properties. Based on the research, the optimal dose of APTES in the solution used for modification was selected: 300 mM of APTES. In this study, the impact of annealing at temperatures up to 500 °C was also examined. The main goal of this study is to evaluate the antibacterial properties of APTES-modified TiO_2_ (calcined in the range of 200–500 °C) against the co-cultures of *Escherichia coli* (ATCC 29425) and *Staphylococcus epidermidis* (DSM 1798).

## 2. Results and Discussion

### 2.1. Characterization of Materials

**Figure 1:**
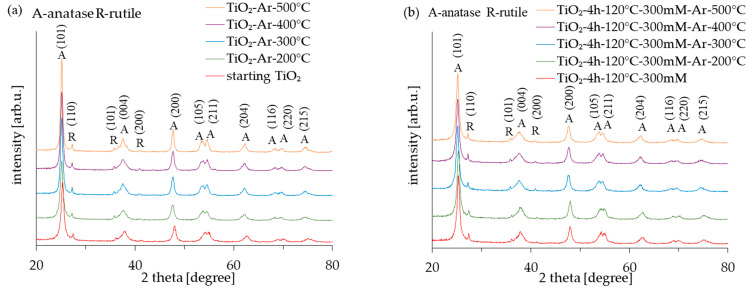
X-ray diffraction patterns of starting TiO_2_, reference calcined photocatalysts (**a**) and APTES/TiO_2_ nanomaterials (**b**).

The diffraction patterns of all the examined photocatalysts (both reference and APTES/TiO_2_) obtained from the XRD analysis results are shown in Figure 1. In turn, the crystallite size and anatase phase content are summarized in Table 1. The primary phase of all the samples is anatase (regardless of the annealing temperature or by APTES modification). According to Figure 1, the XRD peaks for 25.3, 37.8, 48.1, 53.9, 55.1, 62.7, 68.9, 70.3, and 75.1° correlate to the (101), (004), (200), (105), (211), (204), (116), (220), and (215) anatase reflection indexed by JCPDS 01-070-7348. As was presented in Table 1, the amount of anatase in all photocatalysts was approximately 95–96%. Additionally, some peaks (located at 27.4, 36.0, and 41.2°, corresponded to (110), (101), and (111) indexed by JCPDS 01-076-0318, characteristic for the rutile phase, were observed. The presence of rutile in samples results from obtaining raw TiO_2_ (caused by adding the rutile nuclei during the raw TiO_2_ pulp production process using the sulphate method) [31]. The modification of photocatalysts in the temperature range from 200 °C to 500 °C did not cause the transformation of the anatase-to-rutile phase. This is typical because anatase transforms into a rutile phase above 600 °C [32]. The anatase reflexes developed and are more intense (especially in the case of unmodified reference materials), indicating that the non-crystalline amorphous phase transformed into the anatase phase. However, the observed changes were relatively small and, therefore, the presented XRD patterns of TiO_2_ materials are very similar, following the results obtained by Klaysri et al. [26].

The average crystallite size of the APTES-modified samples was estimated at approx. 14–15 nm. On the other hand, the crystallite size of the reference materials increased with the calcination temperature (from 14 nm for starting TiO_2_ to 22 nm for TiO_2_-Ar-500 °C). Generally, the average crystallite size of the samples heated at the same temperature was smaller for APTES/TiO_2_ than for unmodified reference materials. According to the literature [33,34], adding silicon could effectively inhibit the growth of TiO_2_ particles during the calcination process.

**Figure 2:**
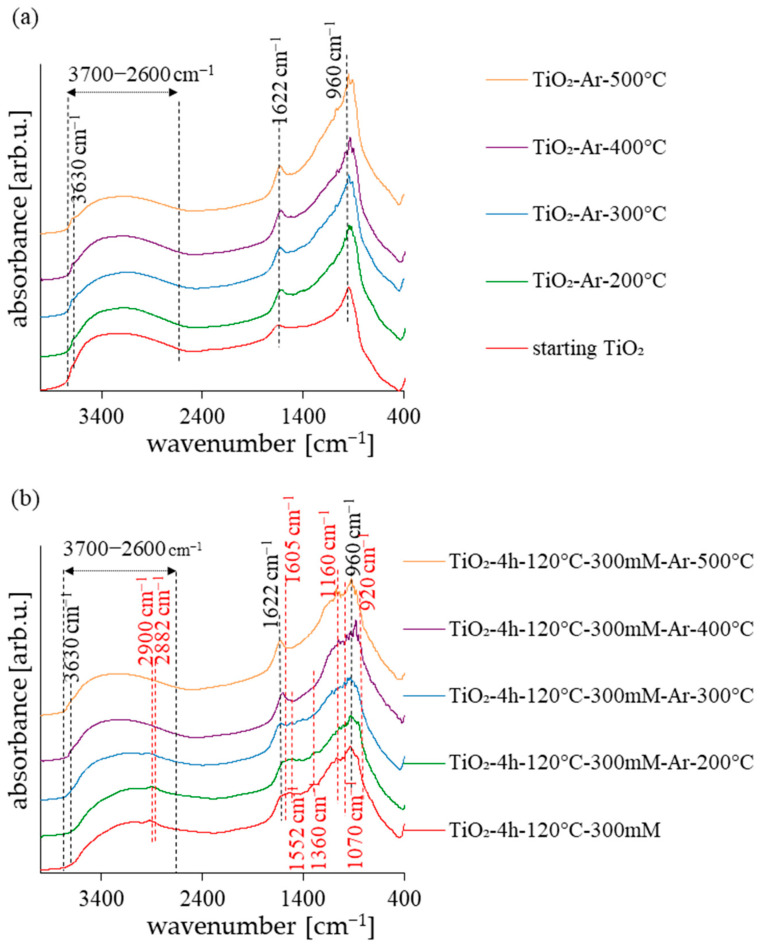
Diffuse reflectance Fourier transform infrared spectra of starting TiO_2_, reference calcined photocatalysts (**a**) and APTES/TiO_2_ nanomaterials (**b**).

The FT-IR analysis has been conducted to illustrate the functional groups of the surface of obtained photocatalysts. The DRIFT spectra of the starting TiO_2_ and reference samples were presented in Figure 2a and the spectra of the APTES-modified samples were shown in Figure 2b. In all the presented spectra, the characteristic peaks for TiO_2_-based photocatalysts can be observed. The broad peak around 3700–2600 cm^−1^ and the narrow band at 1622 cm^−1^ correspond to O–H stretching vibration and the molecular water bending mode, respectively [35,36]. The samples also presented the low-intensity peak at 3630 cm^−1^, attributed to the stretching mode of various free –OH groups. In turn, the peak that can be observed at 960 cm^−1^ is attributed to the self-absorption of titanium (Ti^4+^) [37]. According to Figure 2b, the new bands at 2926, 2870, 1605, 1386, 1155, 1070, and 920 cm^−1^ were observed for the APTES-modified photocatalysts. Two small peaks located around 2926 cm^−1^ and 2870 cm^−1^ are attributed to the asymmetric and symmetric stretches of −CH_2_ groups on the alkyl chain [38,39]. The band at around 1605 cm^−1^ is assigned to NH bending vibration of primary amine. The peaks around 1386 cm^−1^ indicate the presence of a C−N bond [40]. The Si−O−Si stretching vibrations and Si–O–C stretching mode were attributed to the peak values at 1155 cm^−1^ and 1070 cm^−1^, respectively [38,41,42]. The peak at around 920 cm^−1^ suggests that the condensation reaction occurred between silanol and the surface –OH groups [39]. As shown in Figure 2b, some bands in APTES-modified photocatalysts (attributed to alkyl groups, –NH_3_^+^, and C–N bonds) faded with the increasing calcination temperature. As confirmed in previous work, these groups were not permanently attached to the TiO_2_ surface and an increase in the annealing temperature led to the destruction of these bonds [43].

The decomposition of N- and C-containing functional groups were also confirmed using elemental analysis. The existence of carbon and nitrogen in the APTES-modified samples is shown in Table 1. It has been observed that the amount of carbon and nitrogen decreased with the increasing annealing temperature of the samples. The presence of nitrogen (0.18 wt.%) in starting TiO_2_ was due to the preliminary modification of raw material [31]. In the unmodified reference samples, the amount of nitrogen fell below the detection level of the device.

The BET surface area and pore volume of photocatalysts were summarized in Table 2. As expected, as the modification temperature increased, the BET surface area and total pore volume of the reference materials decreased (from 207 m^2^/g and 0.326 cm^3^/g for the starting TiO_2_ to 75 m^2^/g and 0.223 cm^3^/g for TiO_2_-Ar-500 °C). In the case of APTES-modified photocatalysts, a different relationship was observed. The BET surface area of TiO_2_-4 h-120 °C-300 and TiO_2_-4 h-120 °C-300 mM-Ar-200 °C did not practically change (125 and 126 m^2^/g, respectively). In turn, further increasing the modification temperature increased the specific surface area of samples to approx. 155–165 m^2^/g. The increase in the specific surface area and the total pore volume of the samples prepared at 300–500 °C could be explained by the decomposition of APTES molecules. Sienkiewicz et al. [43] noted that, with the increase in the modification temperature, the APTES molecules started degrading and both the external surface of TiO_2_ and pores could be unblocked.

The N_2_ adsorption–desorption isotherms of all the examined samples were shown in Supplementary Materials as Figure S1. According to the IUPAC classification, the presented isotherms were identified as typical type IV specific for adsorption on the mesoporous materials. All the isotherms also presented the same H3 hysteresis loop.

**Figure 3:**
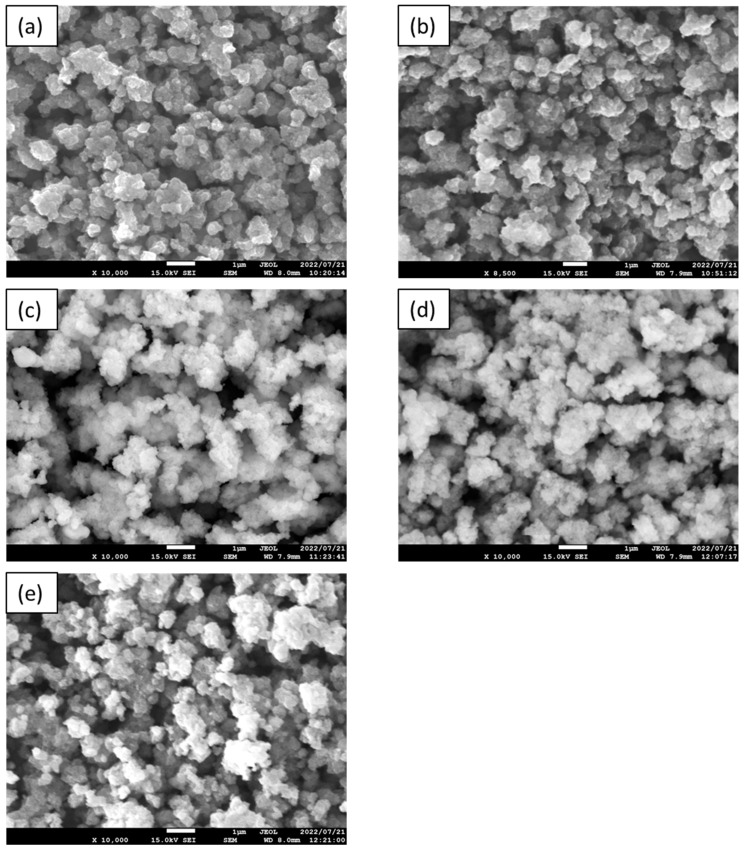
The SEM images of TiO_2_-4 h-120 °C-300 mM (**a**), TiO_2_-4 h-120 °C-300 mM-Ar-200 °C (**b**), TiO_2_-4 h-120 °C-300 mM-Ar-300 °C (**c**), TiO_2_-4 h-120 °C-300 mM-Ar-400 °C (**d**), and TiO_2_-4 h-120 °C-300 mM-Ar-500 °C (**e**).

The SEM images of the APTES-modified samples are presented in Figure 3. The annealing of TiO_2_-4 h-120 °C-300 at temperatures ranging from 200–500 °C did not cause significant changes in its morphology. All the modified photocatalysts presented agglomerated, homogenous, and well-defined nanoparticles.

**Figure 4:**
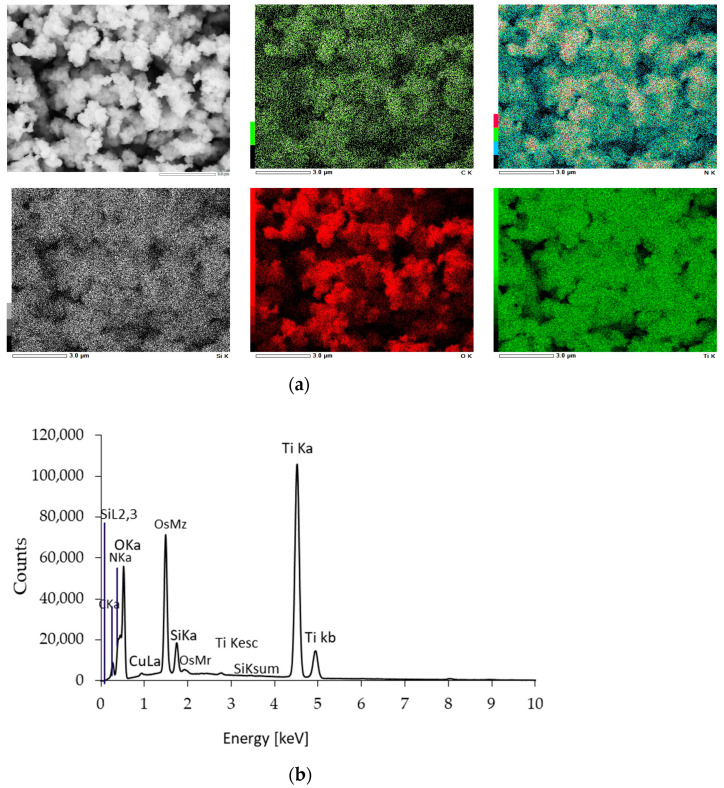
EDS mappings (**a**) and EDS spectrum (**b**) of the exemplary TiO_2_-4 h-120 °C-300 mM-Ar-300 °C.

The distribution of the elemental composition of the APTES-modified photocatalysts was confirmed using EDS mapping analysis and presented in Figure 4 (for TiO_2_-4 h-120 °C-300 mM) and Supplementary Materials as Figures S2–S5 (for the remaining APTES-modified photocatalysts). The results of the EDS mapping analysis confirmed the presence of Ti, O, N, C, and Si. All the examined elements were also uniformly dispersed on the TiO_2_ surface. The Si content (Table 1) was from 2.02 (TiO_2_-4 h-120 °C-300 mM-Ar-400 °C) to 2.51 (TiO_2_-4 h-120 °C-300 mM-Ar-500 °C).

Modifying the TiO_2_ using APTES has also led to the change of surface charge of the samples. As was shown in Table 1, the APTES-modified TiO_2_ nanoparticles were characterized by a more positive zeta potential value. As was reported previously [30,40], the cationic amino groups from the APTES bonded to the TiO_2_ surface and the surface charge of modified materials became more positive. It was also observed that the zeta potential values decreased as the temperature of the sample modification increased. An increase in the modification temperature hindered the binding of positively charged amino groups to the TiO_2_ surface (reflected in the DRIFT measurements and elemental analysis of carbon and nitrogen). As an effect, the modified TiO_2_ presented a less positive zeta potential value. Similar findings were also presented in our previous work [43].

### 2.2. Antibacterial Activity of Photocatalysts

**Figure 5:**
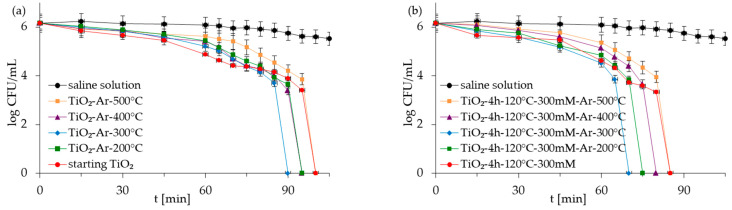
Inactivation of monoculture of *E. coli* in the presence of starting TiO_2_, reference calcined photocatalysts (**a**) and APTES/TiO_2_ samples (**b**) under artificial solar light irradiation.

**Figure 6:**
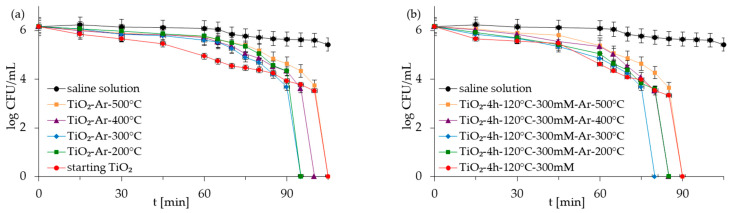
Inactivation of monoculture of *S. epidermidis* in the presence of starting TiO_2_, reference calcined photocatalysts (**a**) and APTES/TiO_2_ samples (**b**) under artificial solar light irradiation.

**Figure 7:**
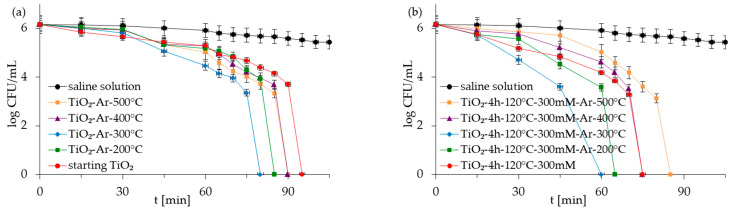
Inactivation of *E. coli* in co-culture of bacteria in the presence of starting TiO_2_, reference calcined photocatalysts (**a**) and APTES/TiO_2_ samples (**b**) under artificial solar light irradiation.

**Figure 8:**
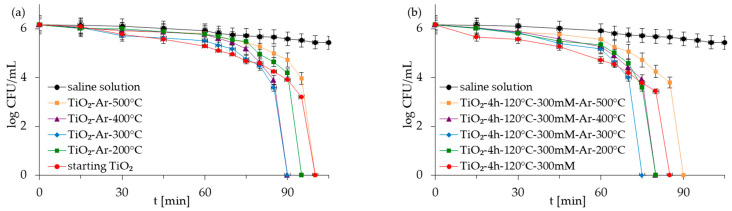
Inactivation of *S. epidermidis* in co-culture of bacteria in the presence of starting TiO_2_, reference calcined photocatalysts (**a**) and APTES/TiO_2_ samples (**b**) under artificial solar light irradiation.

The antibacterial properties of the APTES-modified photocatalysts and reference materials were evaluated for two types of model bacteria: Gram-negative *E. coli* and Gram-positive *S. epidermidis* in monocultures and co-culture forms. The control experiments carried out under dark conditions demonstrated that all the examined photocatalysts did not reduce *E. coli* and *S. epidermidis* populations in experiments with mono- and co-culture bacteria (Supplementary Materials as Figures S6–S9). The presented results indicated that inactivated photocatalysts were not harmful to bacteria. The results for the experiment performed in the absence of the TiO_2_-based samples but under light irradiation (reaction suspension containing only bacteria in saline solution—named saline solution) showed that, alone, artificial solar light (ASL) irradiation also did not affect the viability of bacteria (Figure 5, Figure 6, Figure 7 and Figure 8). Therefore, the inactivation of bacteria due to photolysis can also be neglected.

Figure 5, Figure 6, Figure 7 and Figure 8 show the photocatalytic inactivation of bacteria under ASL irradiation with all the examined nanomaterials. As was presented, both in an experiment with mono- and co-culture bacteria, APTES-TiO_2_ presented better antibacterial properties than unmodified reference materials. The best antibacterial activity was presented for APTES-modified photocatalysts obtained using modification at 300 °C (TiO_2_-4 h-120 °C-300 mM-Ar-300 °C). The complete inactivation of *E. coli* with this sample was achieved after 70 min for mono- and 60 min for co-culture bacteria. In the case of *S. epidermidis*, the time of total inactivation amounted to 80 and 75 min for mono- and co-culture bacteria, respectively.

**Figure 9:**
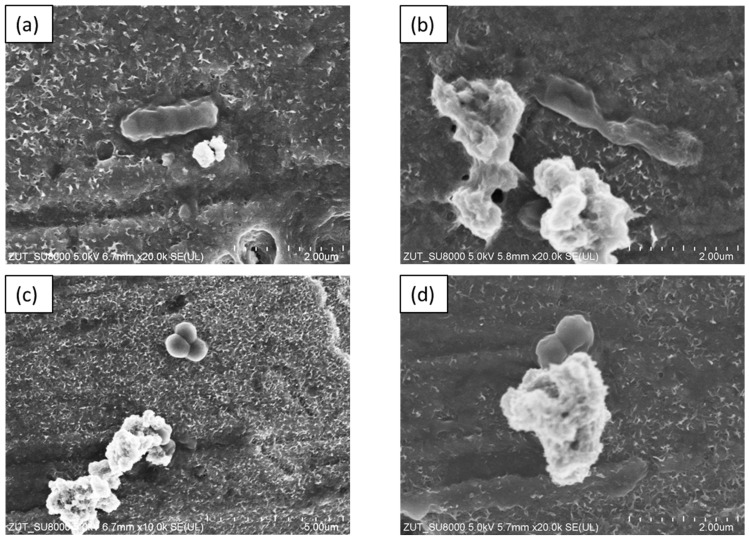
SEM images of bacteria *E. coli*: before (**a**) and after 60 min (**b**) and *S. epidermidis*: before (**c**) and after 60 min (**d**) of photocatalytic process in the presence of TiO_2_-4 h-120 °C-300 mM-Ar-300 °C under ASL irradiation.

The SEM images presented in Figure 9 demonstrate the morphology of bacterial cells in the presence of TiO_2_-4 h-120 °C-300 mM-Ar-300 °C at the beginning and after 60 min of the photocatalytic process. As observed, the bacterial cells presented a regular shape at the beginning of the photocatalytic process. The SEM images registered after 60 min revealed that the surface of the bacteria cells had undergone significant changes. The *E. coli* (Figure 9b) cell was destroyed and lost its characteristic morphology. In the case of *S. epidermidis* (Figure 9d), unevenness in the cell wall can be observed. Additionally, cell wall disruption is visible at the point of contact between the *S. epidermidis* and the photocatalyst.

**Figure 10:**
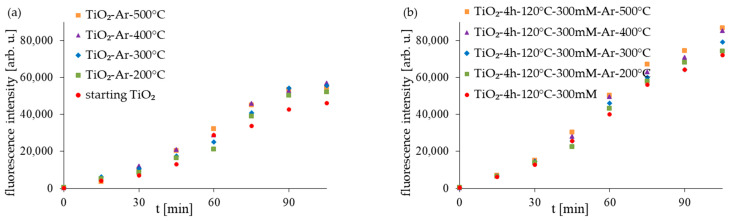
The amount of generated 2-hydroxyterephthalic acid expressed as the peak area of the fluorescent product during ASL irradiation with starting TiO_2_, reference calcined photocatalysts (**a**) and APTES/TiO_2_ samples (**b**).

The good antibacterial properties of APTES-modified photocatalysts can be attributed to various features. One factor affecting photocatalytic activity is the crystal phase (approx. 96% of anatase for tested samples). Many studies confirm that the anatase phase is characterized by higher photocatalytic activity and that photocatalysts mostly containing this crystalline form present the highest antimicrobial properties [44,45]. Another factor is the small crystallite size (14–15 nm). Generally, the most preferred are photocatalysts characterized by small particles (around 11–15 nm or smaller). Many studies confirmed that small particles presented better antimicrobial activity and could cause more damage in bacterial cells [46,47,48,49]. The higher antibacterial activity of APTES-modified TiO_2_ materials was also strongly related to the amount of hydroxyl radicals (^•^OH) produced on photocatalysts’ surfaces (Figure 10). In order to assess the amount of (^•^OH) produced on the sample surface, the fluorescence technique using terephthalic acid was used. In this method, terephthalic acid, which easily reacts with ^•^OH radicals, produces a highly fluorescent product, 2-hydroxyterephthalic acid. The intensity of the peak attributed to 2-hydroxyterephtalic acid is known to be proportional to the amount of formed OH radicals [50]. As was presented in Figure 10, with increasing the ASL illumination time in suspensions containing particular photocatalysts, a gradual increase in the fluorescence intensity is observed, which, in turn, we can attribute to the amount of OH radicals produced on the samples’ surfaces. The APTES-modified photocatalysts generated more hydroxyl radicals than the starting TiO_2_ and reference unmodified materials. However, in the group of the APTES-modified materials, the amount of ^•^OH slightly increases with the increasing modification temperature. Our previous studies on APTES-modified TiO_2_ [29,30] also showed that the high amount of hydroxyl radicals generated from the surface of the APTES-modified photocatalysts is one of the main factors that led to faster bacteria oxidization and, finally, their death.

A thorough analysis of the obtained results has shown that the best antibacterial properties of TiO_2_-4 h-120 °C-300 mM-Ar-300 °C result from several factors’ actions. In addition to those mentioned earlier, in this case, the two most important that cause the enhancement of antibacterial activity could be the large surface area and the positive charge (zeta potential) of the samples. After APTES modification, the surface of the silanized samples becomes more positive. Microorganisms such as bacteria carry a negative charge (in the case of *E. coli* was approx. −44.2 mV and *S. epidermidis* was approx. −42.3 mV). An “electromagnetic” attraction between the negatively charged bacteria cells and the positively charged surface of APTES-TiO_2_ led to closer contact and faster inactivation.

Other factors that play a crucial role in photocatalytic activity are surface area and porous volume. As generally known, a larger surface area can provide more active sites and, therefore, enhance the photocatalytic activity [48,49]. The BET surface area and pore volume of TiO_2_-4 h-120 °C-300 mM-Ar-300 °C are high (S_BET_ = 155 m^2^/g, V_total_ = 0.278 cm^3^/g), which could significantly favor the photocatalytic inactivation of bacteria.

**Figure 11:**
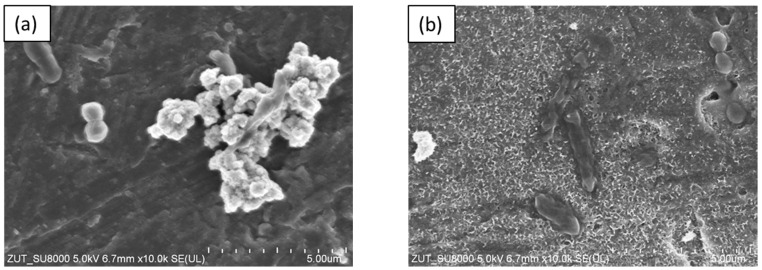
SEM images of bacteria co-culture after 60 min of photocatalytic process in the presence of TiO_2_-4 h-120 °C-300 mM (**a**) and TiO_2_-4 h-120 °C-300 mM-Ar-300 °C under ASL irradiation (**b**).

Additionally, it was observed that photocatalytic inactivation of the co-cultures of two bacteria are faster than those noted for the mono-cultures of bacteria. Rincón and Pulgarin [28] similarly observed this and explained the competition between the two bacteria species for available nutrients. In our case, the experiment was performed in a sterile saline solution with no nutritional properties for bacteria. Moreover, the bacteria were washed to remove the growth medium’s residual. Therefore, we assume that, in our case, this factor may have a small impact on the difference in bacteria inactivation. Comparing the inactivation graphs for mono- and co-cultures of bacteria (Figure 5, Figure 6, Figure 7 and Figure 8), a change in the *E. coli* inactivation curve was observed. According to the literature, a mechanism of bacteria destruction in the presence of TiO_2_ base photocatalysts proceeded in two stages [51]. In the first stage, the microorganisms could trigger self-defense and self-repair mechanisms. Therefore, the number of live bacteria in the reaction solution may remain high. In turn, in the second stage, the mechanisms, as mentioned earlier, are overwhelmed. Highly reactive radicals generated during the photocatalytic process led to disturbances and damage in bacteria cells. Leakage of the intracellular cytoplasmic component may also occur. As a result of this, fast bacteria inactivation was observed. In the case of experiments with monocultures of bacteria (Figure 5 and Figure 6), the second stage started at about 45 min of the photocatalytic process. In an experiment with bacteria co-culture (Figure 7 and Figure 8), this stage began faster—at about 30 min. Since a reaction mixture contains both species of bacteria, the number of each is approx. 0.75 × 10^6^ CFU/mL. *E. coli* inactivation may have occurred first because these bacteria, according to the cell wall structure (the thin cell wall of Gram-negative bacteria), are more susceptible to photocatalytic oxidation. In addition, the number of *E. coli* is less than 1.5 × 10^6^ CFU/mL (considered the optimum amount of bacteria in photocatalytic inactivation tests), which could further contribute to faster inactivation of the bacteria. Then, as the amount of live *E. coli* decreases, *S. epidermidis* is exposed to more significant free radical attack and their faster inactivation also occurs. The SEM images can partially confirm this hypothesis. In Figure 11, the SEM images of the bacterial co-culture after 60 min of the photocatalytic process in the presence of TiO_2_-4 h-120 °C-300 mM-Ar-200 °C and TiO_2_-4 h-120 °C-300 mM-Ar-300 °C under ASL irradiation were shown. In both cases, it can be seen that, after 60 min, the *E. coli* cells were destroyed. *E. coli* have lost their original shape and were significantly damaged. The *S. epidermidis* cells have also shown changes resulting from the treatment of photocatalysts (compared to Figure 9c). However, the damage is not as extensive as in the case of *E. coli*.

## 3. Experimental

### 3.1. Materials and Reagents

As a raw material for photocatalysts production, the crude TiO_2_ slurry, provided by chemical plant Grupa Azoty Zakłady Chemiczne “Police” S.A. (Police, Poland) was used. Before modification, crude TiO_2_ had undergone preliminary modification to reach a pH of 6.8 and the obtained material was denoted as starting TiO_2_. This stage was described in a previous article [31]. As a modifier, 3-aminopropyltriethoxysilane (APTES, C_9_H_23_NO_3_Si, ≥98%) from Merck KGaA (Darmstadt, Germany) was used. As an APTES solvent, ethanol (purity 96%, pure p.a.) purchased from P.P.H. “STANLAB” Sp.J. (Lublin, Poland) was applied.

### 3.2. Preparation of APTES-Modified Photocatalysts

The APTES-functionalized TiO_2_ nanomaterials were obtained using the solvothermal process followed by calcination. For starters, 5 g of startingTiO_2_ was dispersed in 25 mL of the APTES solution with a concentration of 300 mM. Next, the prepared suspension was modified in a pressure autoclave at 120 °C for 4 h, with continuous stirring at 500 rpm. Next, to remove all the remaining chemicals, the mixture was rinsed with ethanol and distilled water and dried in a lab dryer for 24 h at 105 °C. Such a prepared photocatalyst was denoted as TiO_2_-4 h-120 °C-300 mM. Then, the obtained nanomaterial was heated in an argon atmosphere (purity 5.0, Messer Polska Sp. z o.o., Chorzów, Poland). A quartz crucible that contained the appropriate sample was placed in a quartz tube in the center of a GHC 12/900 horizontal furnace (Carbolite Gero, Ltd., Hope, UK). The calcination was carried out in the 200–500 °C range for Δt = 100 °C. Prior to the heating step, argon was passed through the quartz pipe for 0.5 h to eliminate all air in the tube. Next, the furnace was heated up to the specified temperature. The thermal modification was conducted for 4 h with an argon flow of 180 mL/min. After that time, the furnace gradually cooled to room temperature. The photocatalysts obtained after the calcination of starting TiO_2_ in the argon atmosphere were called reference materials and denoted as TiO_2_-Ar-T, while the APTES-functionalized TiO_2_ samples gained after heat treatment were labelled as TiO_2_-4 h-120 °C-300 mM-Ar-T, where T means the temperature of calcination.

### 3.3. Structural Characterization

The obtained samples have been subjected to precise characteristics. The presence of distinct functional groups on the surface of tested photocatalysts was confirmed using the spectrometer FT-IR-4200 (number of scans 100, resolution 4.0 cm^−1^, JASCO International Co. Ltd., Tokyo, Japan), fitted with a DiffuseIR accessory (PIKE Technologies, Fitchburg, MA, USA). The crystalline phase identification and crystal structure of the nanomaterials were performed using the X-ray diffraction analyses (XRD) carried out with a PANalytical Empyrean X-ray diffractometer (Malvern, UK) equipped with Cu Kα radiation (λ = 0.154056 nm). To identify the phase composition, the PDF-4+2014 International Centre for Diffraction Data database (for anatase: 04-002-8296 PDF4+ card, and for rutile: 04-005-5923 PDF4+ card) was used. The crystallite size was determined according to Scherrer’s formula [52]. The Brunauer–Emmett–Teller (BET) specific surface area and pore volume were determined using nitrogen adsorption using the QUADRASORB evoTM Gas Sorption analyzer (Anton Paar GmbH, Graz, Austria). Before measurements, all the photocatalysts were degassed for 12 h at 100 °C under a high vacuum prior to measurements to eliminate all the remaining contaminants on the tested samples’ surfaces. The single-point value determined the total pore volume from the nitrogen adsorption isotherms at relative pressure p/p0 = 0.99. The micropore volume (V_micro_) was determined using the Dubinin–Radushkevich method. In turn, the mesopore volume was estimated as the difference between V_total_ and V_micro_. The elemental contents of nitrogen and carbon in the TiO_2_ samples were measured using a CN 628 elemental analyzer (LECO Corporation, St. Joseph, MI, USA). The certified soil standard (Elemental Microanalysis Ltd., Okehampton, UK), containing 0.043 wt.% ± 0.01 of nitrogen and 0.46 wt.% ± 0.15 of carbon, was utilized to prepare the calibration curve. The error range for the measurements was maximally ±0.1%. The zeta potential values of nanoparticles were determined using a ZetaSizer NanoSeries ZS (Malvern PANalytical Ltd., Malvern, UK). The samples were dispersed in sterile H_2_O, sonicated for 30 min, and then subjected to analysis. All the experiments were carried out tenfold and the data were depicted as averages (while the difference between the considered measurements was not greater than 10%). The morphology of the APTES-modified samples was observed using Field Emission Scanning Electron Microscopy (FE-SEM, JSM 7600F, Jeol Ltd., Tokyo, Japan). In turn, the morphology of the bacteria cells was evaluated using a Hitachi SU8020 Ultra-High Resolution Field Emission Scanning Electron Microscope (Hitachi Group, Japan). The surface chemical analysis of the APTES-modified samples was performed using energy dispersive X-ray spectroscopy (EDS) analyzed using an EDS system combined with FE-SEM (JSM-7600F, Jeol Ltd., Tokyo, Japan). The analysis of the hydroxyl radicals (^•^OH) formation on the photocatalyst surface under artificial solar light irradiation was determined using the fluorescence technique with terephthalic acid (Acros Organics B.V.B.A, Geel, Belgium) according to the method described in our previous work [30]. The analysis was performed using a Hitachi F-4500 fluorescence spectrophotometer (Hitachi Group, Tokyo, Japan).

### 3.4. Preparation of Microorganisms and Antibacterial Activity Tests

The studies used Gram-negative *Escherichia coli* (ATCC 29425, LGC Group, Kielpin, Poland) and Gram-positive *Staphylococcus epidermidis* (DSM 1798, DSMZ, Braunschweig, Germany) as microorganisms. The sterile nutrient broth (Biomaxima S.A., Poland) and brain infusion broth (Biomaxima S.A., Lublin, Poland) were inoculated with *E. coli* and *S. epidermidis*, respectively, and incubated for 24 h at 37 °C. Next, the bacteria cells were harvested using centrifugation (at 4000 rpm for 10 min) and washed with 0.9% saline solution (Merck, Germany) to remove the residues of the growth broth. Finally, the bacteria concentration was adjusted to approx. 1.5 × 10^7^ CFU/mL using spectrometric measurements (optical density measurement, OD600).

### 3.5. Antibacterial Activity Test

All the glassware used in the experiment was washed with distilled water and thermally sterilized at 150 °C for 24 h before each experiment.

The antibacterial tests were carried out in 150 mL glass beakers used as the reactor. The experiments were conducted both for mono- and co-cultures of bacteria. In the experiments with monoculture, 10 mL of the bacterial suspension (1.5 × 10^7^ CFU/mL) was transferred to the beaker containing 90 mL of saline solution (0.9%) and 10 mg of the appropriate photocatalyst. For the experiments with mixtures of *E. coli* and *S. epidermidis*, 5 mL of each bacteria suspension was transferred into the same beaker. Finally, in each experiment, the bacteria concentration was approx. 1.5 × 10^6^ CFU/mL and the photocatalyst concentration amounted to 0.1 g/L. The reaction suspension was irradiated from above using artificial solar light, denoted as ASL, for 90 min. The employed ASL source comprised a 300 W light bulb (OSRAM Ultra Vitalux, OSRAM GmbH, Munich, Germany) with a radiation intensity of 9.0 W/m^2^ in the spectral range from 300 to 2800 nm and 258.1 W/m^2^ in the spectral range from 280 to 380 nm. The distance between the beaker and the light source was fixed at approx. 40 cm. The bacteria inactivation process was conducted at room temperature and the temperature of the reaction suspension amounted to approx. 25 °C. The light source’s emission spectra and radiation intensity were presented in Supplementary Materials as Figure S10. Before the inactivation process, the ASL lamp was preheated for 30 min to obtain a stable light intensity.

The antibacterial activity of the photocatalysts was evaluated using the standard plate count method. The reaction suspension (1 mL) was initially measured every 15 min and then every 5 min. Subsequently, the mixture was diluted to a particular concentration (10^−3^ mL) and then 250 μL of the mixture was uniformly coated on the Petri dish’s surface. First, *E. coli* was spread over the surface of Lactose Agar TTC with Tergitol 7 (BTL Sp. z o.o., Łódź, Poland). For the *S. epidermis*, Mannitol Salt Lab Agar (BioMaxima S.A., Lublin, Poland) was used. Next, the plates were incubated at 37 °C for 24 h (with *E. coli*) and 48 h (with *S. epidermidis*) and the number of colonies was counted by using a bacterial colony counter LKB 2002 (POL-EKO-APARATURA Sp.j., Wodzisław Śląski, Poland). Simultaneously, control experiments under dark conditions and without photocatalysts (only ASL light) were also performed. All the experiments were carried out in triplicate and presented as the average, including standard deviation.

## 4. Conclusions

The photocatalysts were obtained by modification of TiO_2_ using APTES with a solvothermal process (at 120 °C) followed by calcination (200–500 °C). The calcination of the APTES-modified TiO_2_ caused a further improvement in the samples’ antibacterial properties. The received APTES/TiO_2_ were capable of total *Escherichia coli* and *S. epidermidis* inactivation in the experiments performed with mono- and co-cultures of bacteria under artificial solar light irradiation. In addition, for the experiments performed with bacterial co-culture, the bacteria inactivation time was shorter compared to the experiments with bacteria monocultures. The positive surface charge of the photocatalysts and high S_BET_ area could be the main factors responsible for the good antibacterial properties of the APTES-modified photocatalysts, calcined in the range of 200–500 °C.

## Figures and Tables

**Table 1:** Physicochemical properties of starting, reference, and APTES-modified TiO_2_ photocatalysts.

Name Sample	Anatase in Crystallite Phase [%]	Anatase Crystallite Size [nm]	Carbon Content [wt.%]	Nitrogen Content [wt.%]	Silica Content [wt.%] **
Starting TiO_2_	95	14	-	0.18	-
TiO_2_-Ar-200 °C	96	14	-	*	-
TiO_2_-Ar-300 °C	96	18	-	*	-
TiO_2_-Ar-400 °C	95	18	-	*	-
TiO_2_-Ar-500 °C	95	22	-	*	-
TiO_2_-4 h-120 °C-300 mM	96	15	4.11	1.43	2.39
TiO_2_-4 h-120 °C-300 mM-Ar-200 °C	96	15	3.45	1.19	2.30
TiO_2_-4 h-120 °C-300 mM-Ar-300 °C	96	14	2.66	0.69	2.31
TiO_2_-4 h-120 °C-300 mM-Ar-400 °C	96	14	0.81	0.24	2.10
TiO_2_-4 h-120 °C-300 mM-Ar-500 °C	96	15	0.65	0.12	2.51

* beyond the detection level; ** results from EDS mapping analysis.

**Table 2:** Structural parameters of starting, calcined, and APTES-modified TiO_2_ photocatalysts.

Name Sample	S_BET_ [m^2^/g]	V_total(0.99)_ [cm^3^/g]	V_meso(DR)_ [cm^3^/g]	V_micro_ [cm^3^/g]	Zeta Potential δ [mV]
starting TiO_2_	207	0.326	0.072	0.254	+6.83
TiO_2_-Ar-200 °C	166	0.250	0.059	0.191	+12.02
TiO_2_-Ar-300 °C	112	0.288	0.041	0.247	+14.08
TiO_2_-Ar-400 °C	95	0.249	0.060	0.189	+14.74
TiO_2_-Ar-500 °C	75	0.223	0.030	0.193	+15.01
TiO_2_-4 h-120 °C-300 mM	125	0.219	0.047	0.172	+21.66
TiO_2_-4 h-120 °C-300 mM-Ar-200 °C	126	0.205	0.156	0.049	+21.08
TiO_2_-4 h-120 °C-300 mM-Ar-300 °C	155	0.278	0.056	0.222	+20.38
TiO_2_-4 h-120 °C-300 mM-Ar-400 °C	160	0.249	0.060	0.189	+16.53
TiO_2_-4 h-120 °C-300 mM-Ar-500 °C	157	0.266	0.059	0.207	+12.09

## Data Availability

The data presented in this study are available on request from the corresponding author.

## Supplementary Materials

The following supporting information can be downloaded at: https://www.mdpi.com/article/10.3390/molecules28041655/s1. Figure S1: Adsorption–desorption isotherms of starting TiO_2_, reference calcined photocatalysts (a), and APTES/TiO_2_ nanomaterials (b). Figure S2: EDS mapping and EDS spectrum of TiO_2_-4 h-120°C-300 mM. Figure S3: EDS mapping and EDS spectrum of TiO_2_-4 h-120°C-300 mM-Ar-200 °C. Figure S4: EDS mapping and EDS spectrum of TiO_2_-4 h-120°C-300 mM-Ar-400 °C. Figure S5: EDS mapping and EDS spectrum of TiO_2_-4 h-120°C-300 mM-Ar-500 °C. Figure S6: Inactivation of monoculture of *E. coli* in the presence of starting TiO_2_, reference calcined photocatalysts (a) and APTES/TiO_2_ samples (b) under dark conditions. Figure S7: Inactivation of monoculture of *S. epidermidis* in the presence of starting TiO_2_, reference calcined photocatalysts (a) and APTES/TiO_2_ (b) samples under dark conditions. Figure S8: Inactivation of *E. coli* in co-culture bacteria in the presence of starting TiO_2_, reference photocatalysts (a) and APTES/TiO_2_ (b) samples under dark conditions. Figure S9: Inactivation of *S. epidermidis* in co-culture of bacteria in the presence of starting TiO_2_, reference photocatalysts (a) and APTES/TiO_2_ samples (b) under dark conditions. Figure S10: Emission spectra of artificial solar light (ASL).

## Sample Availability

Samples of the compounds are available from the authors.
