# Supplementary material for: Photocatalytic Inactivation of Co-Culture of E. coli and S. epidermidis Using APTES-Modified TiO2
Source: Molecules. 2023 Feb 9;28(4):1655. doi: 10.3390/molecules28041655 (PMC9965180; doi:10.3390/molecules28041655)
Supplement: Supplementary file 1 [file molecules-28-01655-s001.zip › molecules-2167099-supplementary.pdf]

Photocatalytic Inactivation of Co-Culture of *E. coli* and S. *epidermidis* using APTES-Modified TiO_2

Paulina Rokicka-Konieczna [1,2]*, Agnieszka Wanag [1], Agnieszka Sienkiewicz [1], Dylan Shun Izuma [2], Ewa Ekiert [1], Ewelina Kusiak-Nejman [1], Chiaki Terashima [2,3], Atsuo Yasumori [4], Akira Fujishima [3] and Antoni W. Morawski [1]

[1] Department of Inorganic Chemical Technology and Environment Engineering, Faculty of Chemical Technology and Engineering, West Pomeranian University of Technology in Szczecin, Pułaskiego 10, 70-322 Szczecin, Poland;

[2] Department of Pure and Applied Chemistry, Faculty of Science and Technology, Tokyo University of Science, 2641 Yamazaki, Noda, Chiba 278-8510, Japan

[3] Research Center for Space System Innovation, Research Institute for Science and Technology, Tokyo University of Science, 2641 Yamazaki, Noda, Chiba 278-8510, Japan

[4] Department of Materials Science and Technology, Faculty of Advanced Engineering, Tokyo University of Science, 6-3-1 Niijuku, Katsushika-ku, Tokyo 125-8585, Japan

* Correspondence: paulina.rokicka@zut.edu.pl

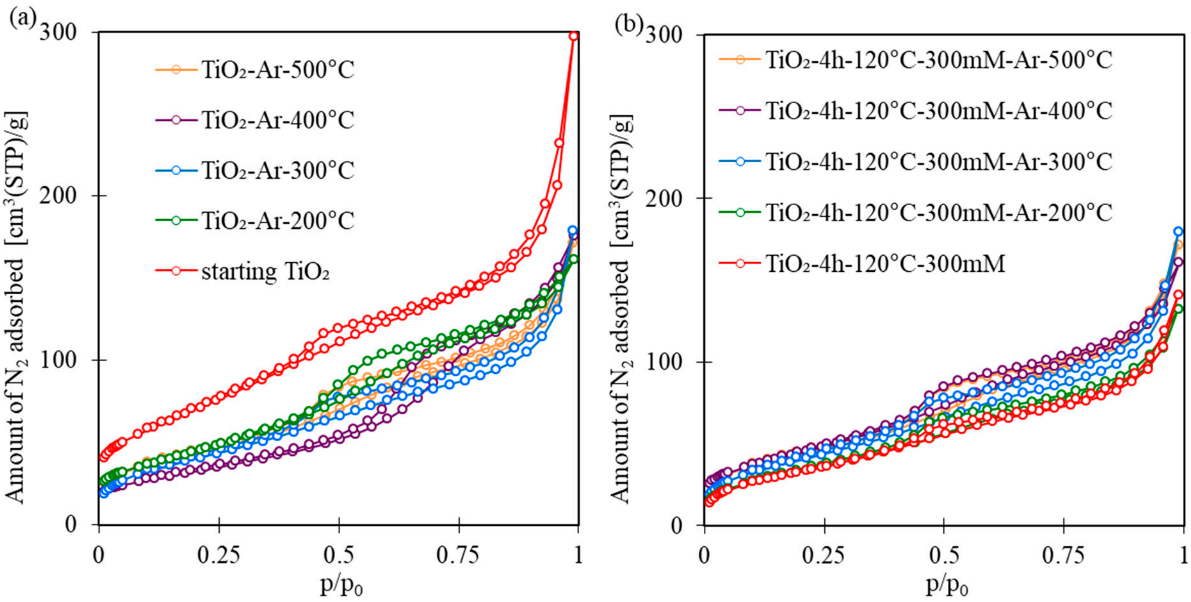

Figure S1. Adsorption-desorption isotherms of starting TiO_2, reference calcined photocatalysts (a), and APTES/TiO_2 nanomaterials (b).

(a)

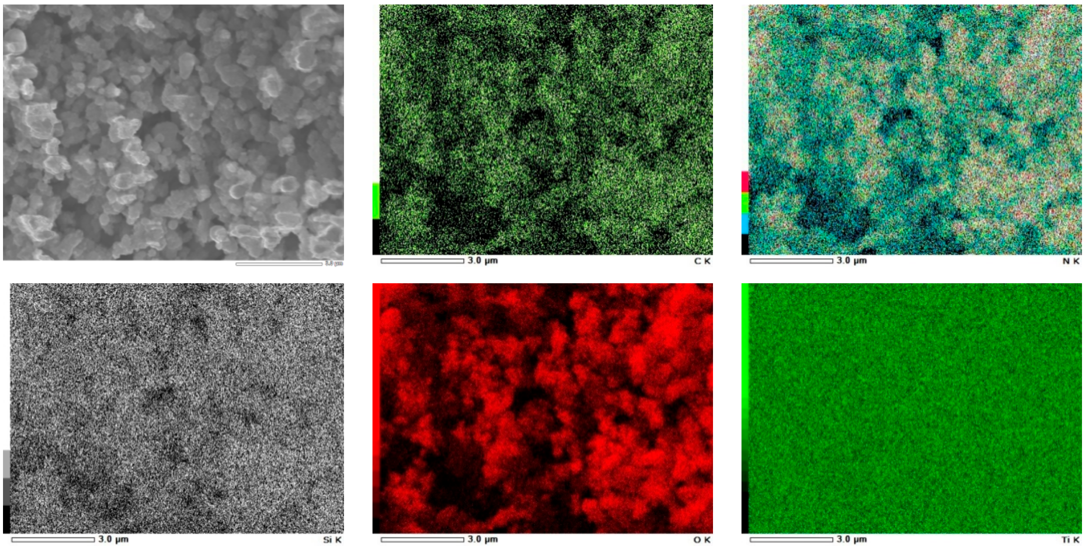

(b)

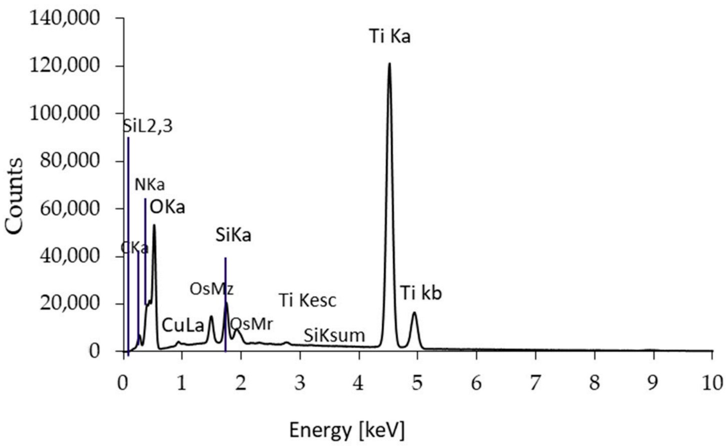

Figure S2. EDS mapping (a) and EDS spectrum (b) of TiO_2-4 h-120°C-300 mM

(a)

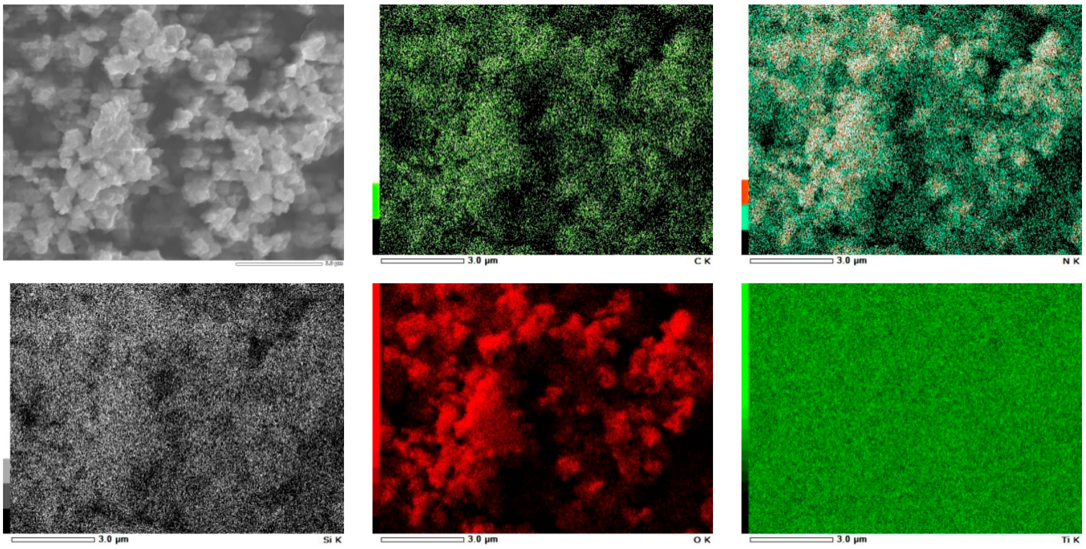

(b)

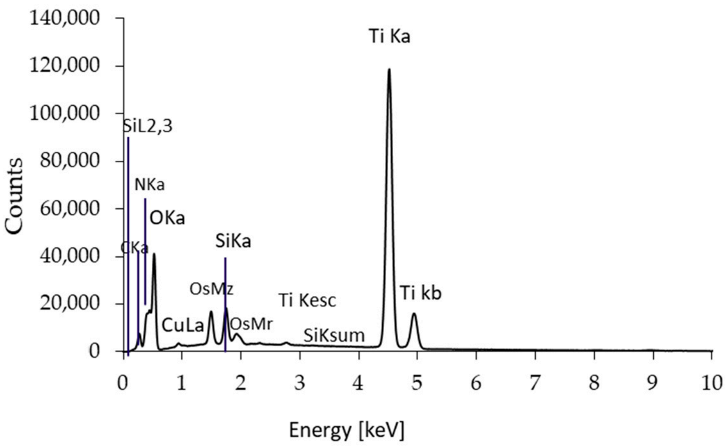

Figure S3. EDS mapping (a) and EDS spectrum (b) of TiO_2-4 h-120°C-300 mM-Ar-200 °C.

(a)

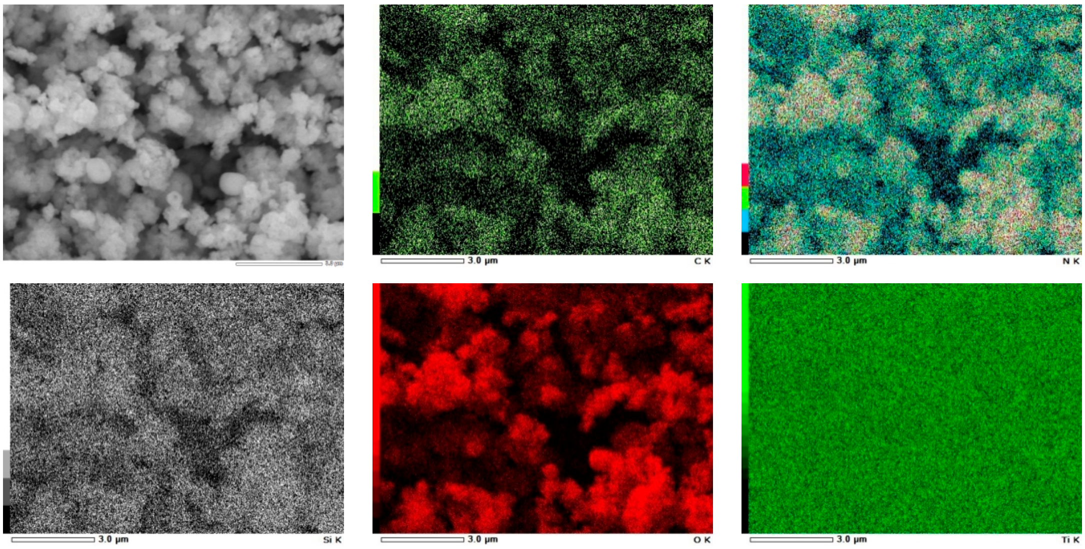

(b)

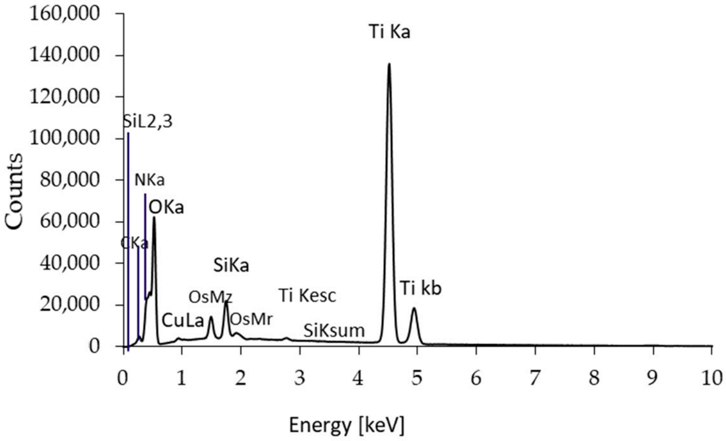

Figure S4. EDS mapping (a) and EDS spectrum (b) of TiO_2-4 h-120°C-300 mM-Ar-400 °C.

(a)

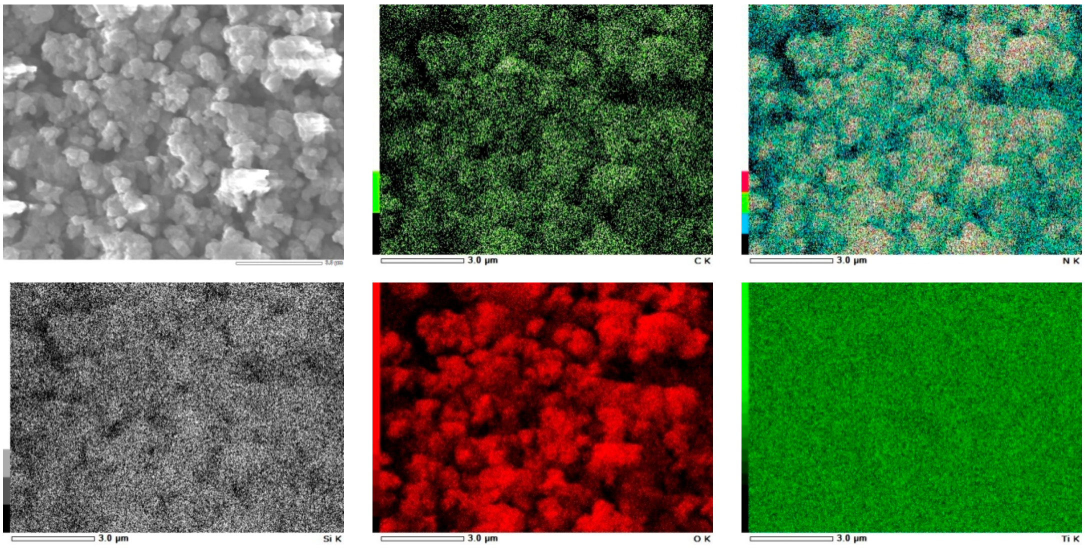

(b)

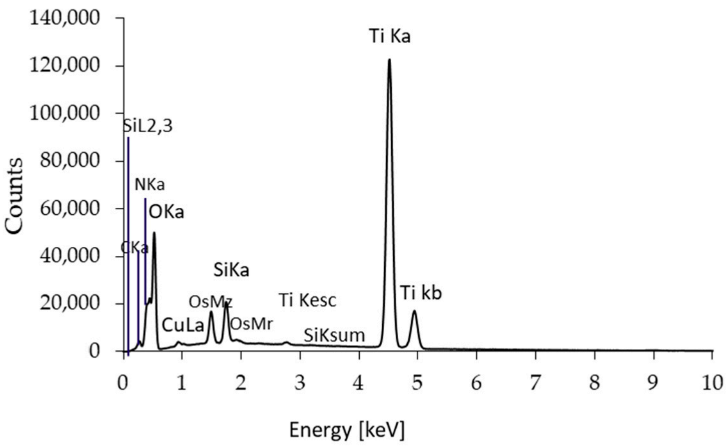

Figure S5. EDS mapping (a) and EDS spectrum (b) of TiO_2-4 h-120°C-300 mM-Ar-500 °C.

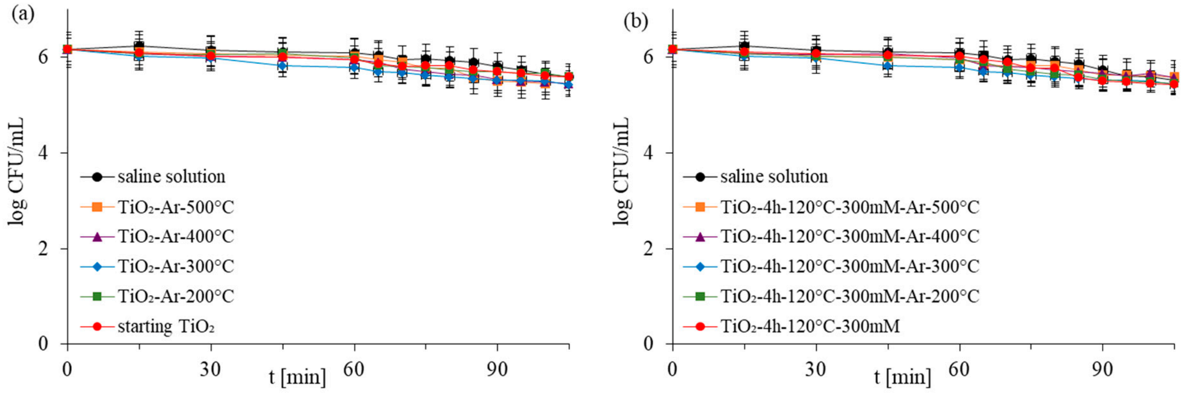

Figure S6. Inactivation of monoculture of *E. coli* in the presence of starting TiO_2, reference calcined photocatalysts (a) and APTES/TiO_2 samples (b) under dark conditions.

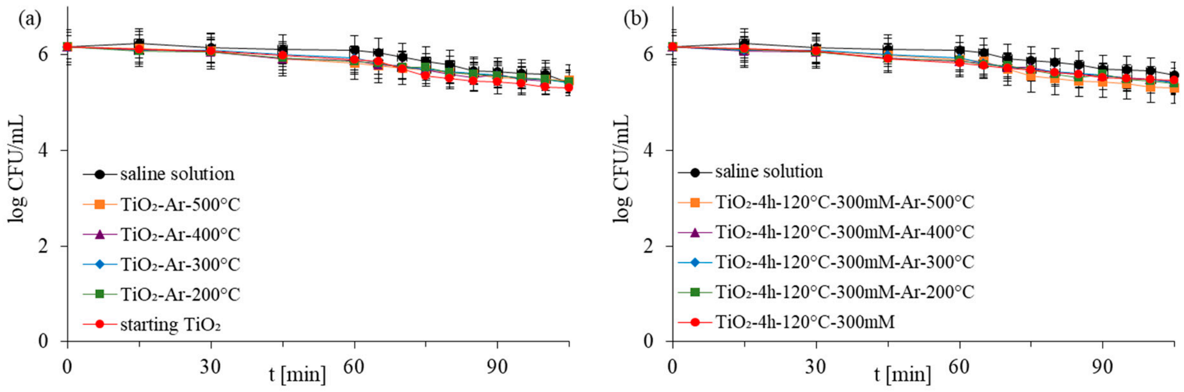

Figure S 7. Inactivation of monoculture of *S. epidermidis* in the presence of starting TiO_2, reference calcined photocatalysts (a) and APTES/TiO_2 (b) samples under dark conditions.

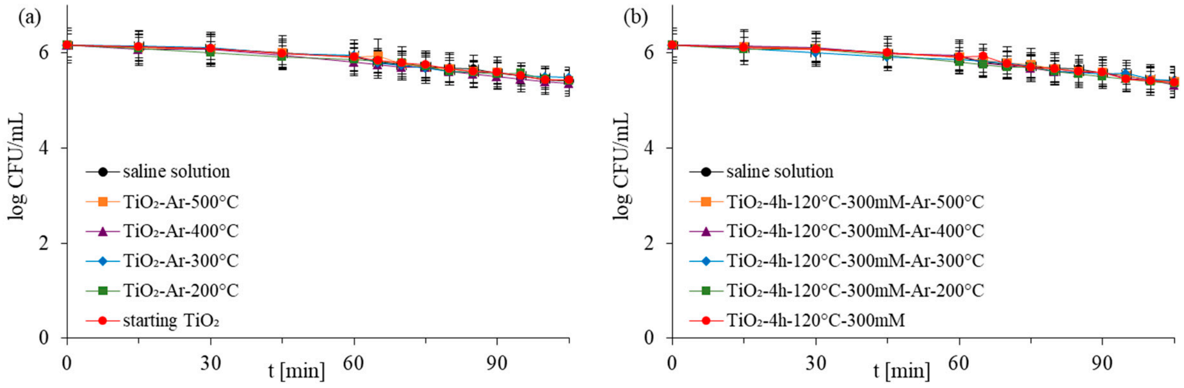

Figure S8. Inactivation of *E. coli* in co-culture bacteria in the presence of starting TiO_2, reference photocatalysts (a) and APTES/TiO_2 (b) samples under dark conditions.

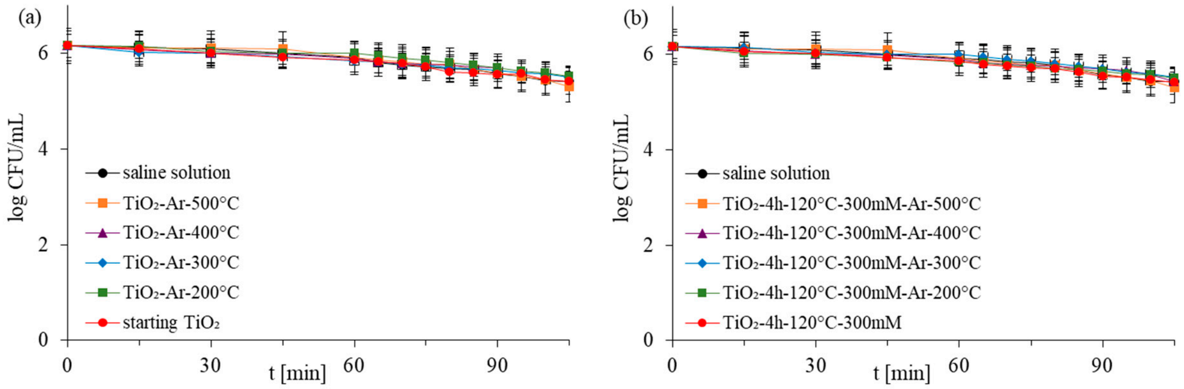

Figure S9. Inactivation of *S. epidermidis* in co-culture of bacteria in the presence of starting TiO_2, reference photocatalysts (a) and APTES/TiO_2 samples (b) under dark conditions.

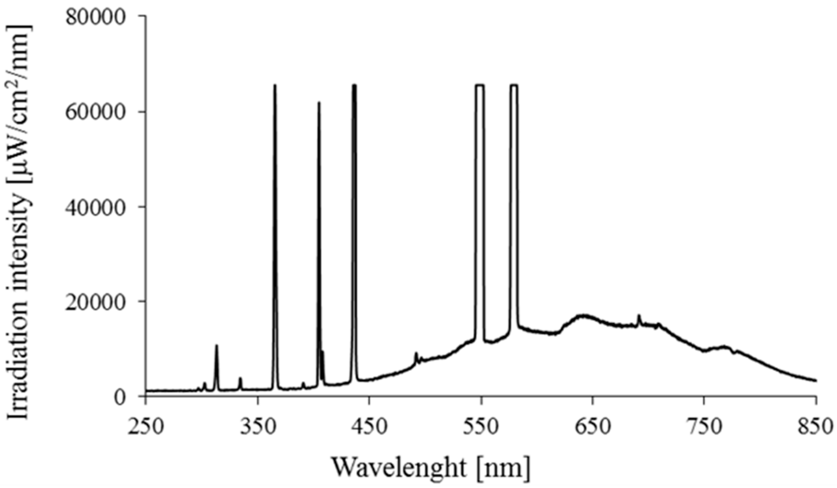

Figure S10. Emission spectra of artificial solar light (ASL).

The employed ASL source comprised a 300 W light bulb (OSRAM Ultra Vitalux, OSRAM GmbH, Munich, Germany) with a radiation intensity of 9.0 W/m^2 in the spectral range from 300 to 2800 nm and 258.1 W/m^2 in the spectral range from 280 to 380 nm.
